# Comparative effectiveness of ambient documentation tools in primary care tool-specific variations in efficiency, documentation burden, and productivity over time

**DOI:** 10.1093/jamiaopen/ooag155

**Published:** 2026-08-11

**Authors:** Lidia Moura, Rebecca G Mishuris, Joshua P Metlay, Mamoon Habib, Jacqueline G You, Katherine L Gallagher, Suzanne Brodney, Meghan L Rieu-Werden, Jennifer S Haas

**Affiliations:** Department of Neurology, Massachusetts General Hospital, Boston, MA 02114, United States; Center for Value-Based Healthcare and Sciences, Massachusetts General Hospital, Boston, MA 02114, United States; Department of Epidemiology, Harvard T. H. Chan School of Public Health, Boston, MA 02115, United States; Harvard Medical School, Boston, MA 02115, United States; Harvard Medical School, Boston, MA 02115, United States; Division of General Internal Medicine, Mass General Brigham, Boston, MA 02115, United States; Harvard Medical School, Boston, MA 02115, United States; Division of General Internal Medicine, Mass General Brigham, Boston, MA 02115, United States; Department of Neurology, Massachusetts General Hospital, Boston, MA 02114, United States; Center for Value-Based Healthcare and Sciences, Massachusetts General Hospital, Boston, MA 02114, United States; Harvard Medical School, Boston, MA 02115, United States; Division of General Internal Medicine, Mass General Brigham, Boston, MA 02115, United States; Division of General Internal Medicine, Mass General Brigham, Boston, MA 02115, United States; Division of General Internal Medicine, Mass General Brigham, Boston, MA 02115, United States; Division of General Internal Medicine, Mass General Brigham, Boston, MA 02115, United States; Harvard Medical School, Boston, MA 02115, United States; Division of General Internal Medicine, Mass General Brigham, Boston, MA 02115, United States

**Keywords:** artificial Intelligence, physician well-being, primary health care, electronic health records, clinical documentation

## Abstract

**Objectives:**

To evaluate the comparative effectiveness of ambient documentation tools (ADTs) with distinct architectures (Tablet-Based Virtual Human–Assisted Ambient [Tool A], EHR-Integrated Ambient [Tool B], and Standalone Ambient [Tool C]) on provider efficiency, documentation burden, and productivity in primary care.

**Materials and Methods:**

We conducted a real-world comparative-effectiveness study of 163 primary care providers in a large integrated health system (January 2024-June 2025), analyzing 59 130 provider-days. Providers who did not adopt an ADT (*n* = 13) were excluded from comparative models. Exposure groups included Tool A (*n* = 65), Tool B (*n* = 68), and Tool C (*n* = 17). Primary outcomes were pajama time (hours of after-hours EHR activity per provider-day), visit closure within 2 days (proportion of encounters signed within 48 hours), proportion of manual note composition, and a composite opportunity score reflecting EHR efficiency (scaled 0-1). Outcomes were derived from daily provider-level EHR logs. Analyses used intention-to-treat and per-protocol frameworks with provider-clustered SEs and month fixed effects.

**Results:**

Compared with Tool B, Tool A was associated with increased pajama time (+0.022 hours of after-hours EHR activity per provider-day), higher manual note composition (+0.046 absolute proportion), lower timely visit closure (−0.120 [95% CI, −0.126,−0.115]), and reduced opportunity scores (−0.035[−0.044,−0.027]). Tool C reduced after-hours work versus Tool B (−0.007 hours per provider-day) and relative to both the reference tool and pre-exposure baseline levels (−0.055 hours per provider-day), but was associated with lower timely visit closure (−0.028[−0.036, −0.020]); opportunity scores were not significantly changed (−0.007[−0.018, 0.003]). Effects for Tool C correspond to approximately 30 fewer annual after-hours hours per provider.

**Conclusion:**

Tool choice materially shapes real-world outcomes, and tool-specific comparative evaluation is essential to guide evidence-based procurement and deployment decisions.

## Background and significance

The electronic health record (EHR) has transformed clinical documentation by centralizing patient data, facilitating communication across care teams, and supporting quality measurement. However, the burden of EHR documentation has been well documented as a source of clinician frustration, after-hours workload (“pajama time”), and burnout.^1–4^ The high proportion of notes composed manually, coupled with delays in closing encounters, reflects inefficiencies that limit time available for direct patient care.^5^

As health systems increasingly deploy ambient documentation tools to address this burden, decision-makers face near-term procurement, renewal, and consolidation choices in a rapidly evolving market, often without rigorous, tool-specific comparative evidence to guide those investments.

To address these challenges, health systems have increasingly turned to scribing workflows, which aim to reduce the need for manual EHR entry by the clinician by capturing, transcribing, and structuring clinical encounters.^6–9^ Two broad categories of solutions exist: (1) human scribes, who listen to encounters and draft clinical notes on behalf of the clinician (either in person or remotely) and (2) AI-enabled ambient documentation tools, which leverage speech recognition and large language models (generative AI) to generate draft clinical notes automatically.^10^

Despite rapid adoption—driven by workforce constraints and the rising cost of human scribes—rigorous, head-to-head evidence comparing tool-specific real-world effects remains sparse, even though these platforms differ substantially in architecture, workflow integration, human involvement, and implementation burden.

At our institution, 3 ambient documentation strategies have been widely deployed (Table 1).

**Table 1.** Comparison of ambient documentation tools: platform features and observed implementation characteristics.

Domain	Tool A—tablet-based, human-assisted (scribble)	Tool B—EHR-integrated ambient tool (DAX)	Tool C—standalone AI tool with later integration (abridge)
**Integration and deployment**	Not EHR-integrated; requires manual transfer (copy/paste) into EHR	Fully integrated within EHR from initial deployment	Initially standalone requiring manual transfer (copy/paste); later integrated into EHR
**Workflow model**	Audio recorded during visit, then transmitted to remote human scribe, then draft note returned hours later for clinician review and upload	Automated ambient capture with near real-time draft note generation directly within EHR	Fully automated AI-driven note generation via external interface; later streamlined with EHR integration
**Timeliness of documentation**	Delayed (typically several hours post-visit)	Near real-time availability within clinical workflow	Near-immediate note generation; early phase required manual transfer, later improved with integration
**Documentation generation approach**	Human-assisted: remote scribe performs transcription and drafting based on audio recording	AI-assisted transcription with clinician review and minor edits	Fully AI-generated documentation with minimal manual drafting
**Verification burden**	High: extensive review and editing required due to human transcription variability	Moderate: requires clinician review with minor edits	Moderate to low: reduced editing required after integration; still requires clinician verification
**Accuracy (observed performance)**	High accuracy with human transcription, but variability and inefficiency in workflow	Variable accuracy, particularly during early adoption	High accuracy reported in mature phase, supporting reduced documentation burden
**Human vs AI involvement**	High human involvement (remote scribes performing transcription and drafting)	Primarily AI-driven with clinician oversight	Fully AI-driven with minimal human intervention
**Documentation characteristics**	Narrative note drafted by human scribe; variable formatting depending on workflow	Structured and semi-structured note integrated within EHR templates	AI-generated note; adaptable format; optimized for efficiency and downstream use
**Language support**	English only	English only (initially)	Multilingual from inception (key differentiator)
**Operational considerations**	Requires coordination with external scribe service; additional workflow steps for upload and verification	Seamless workflow within EHR; minimal disruption to existing processes	Early workflow friction due to manual transfer; later improved usability and efficiency after integration

Features reflect a combination of platform capabilities and observed real-world implementation characteristics within the study setting.

These platforms differ meaningfully in their degree of EHR integration, reliance on human scribes, turnaround time, verification burden, and workflow handoffs. Such architectural and implementation differences are likely to produce divergent effects on documentation efficiency, visit throughput, and after-hours workload. Treating ambient documentation tools as interchangeable may therefore obscure clinically and operationally meaningful variation in real-world impact.

Early reports have suggested potential benefits for reducing pajama time and improving note quality, but results are inconsistent and often limited to single-vendor evaluations.^6^^,^^11–13^ Furthermore, the assumption that ambient documentation solutions are interchangeable may obscure meaningful differences in outcomes across vendors and platform characteristics.^13–15^

Because randomized rollout of enterprise EHR technologies is rarely feasible, observational comparative effectiveness designs are uniquely suited to capture the pragmatic impact of ambient documentation tools as they are deployed under routine clinical conditions. Such real-world evaluations are essential for understanding workflow integration effects, sustained adoption patterns, and vendor-specific performance differences that cannot be readily reproduced in tightly controlled trials.

## Objective

In this study, we conducted a retrospective, provider-day–level cohort analysis spanning 3 years of EHR data across a large integrated health system, focused on primary care clinicians for whom documentation burden has become a critical operational challenge. We evaluated the comparative effectiveness of 3 ambient documentation strategies (Tablet-Based, Virtual Human-Assisted Ambient Tool [Tool A], EHR-Integrated Ambient Tool [Tool B], and Standalone Ambient Tool [Tool C]) on pajama time, manual note composition, visit closure, and opportunity score.

Our goal was to generate pragmatic, tool-specific comparative effectiveness evidence reflecting real-world workflow integration and sustained use. To ensure rigorous comparison, we used the EHR-Integrated Ambient Tool as the reference platform; excluded non-adopters from comparative analyses while retaining them for descriptive context; examined outcomes under both intention-to-treat (ITT) and per-protocol (PP) frameworks; and conducted sensitivity analyses across 3-, 6-, 9-, and 12-month post-initiation windows.

This study provides one of the first real-world, tool-specific comparative effectiveness evaluations of ambient documentation platforms, addressing a critical evidence gap and informing health system decision-making around documentation burden, efficiency, and productivity.

## Methods

### Study design and setting

We conducted a retrospective observational cohort study using detailed, provider-day–level EHR data from January 1, 2024, through June 30, 2025. The study took place within Mass General Brigham (MGB),^16^ a large integrated academic health system in the United States. The system includes family medicine, internal medicine, and primary care practices staffed by physicians and advanced practice clinicians (APCs). Ambient documentation strategies were rolled out in phases across practices beginning in early 2024, providing natural variation in exposure. Within primary care, ambient tools were offered via 2 complementary pathways. First, a legacy tool was replaced with its next-generation version (Tool A) through a rolling, practice-by-practice migration: practice medical directors communicated the change; all active users of the legacy tool in each practice were converted to the new version (no option to retain the legacy tool), and new adopters were offered the new version only. Clinicians could discontinue the new version and move to an alternative ambient platform available through the program, but could not revert to the legacy tool. Second, in a concurrent real-world head-to-head pilot, all primary care clinicians received a broadcast invitation to join an electronic interest list; enrolled clinicians were assigned to 1 of 2 alternative platforms (Tools B and C), with allocation constrained by basic technical compatibility (eg, mobile OS/version). Clinicians could subsequently request a platform change if needed. The vendors included IKS Health, Abridge, and Nuance DAX Copilot.

Because randomized rollout of enterprise EHR technologies is rarely feasible, we adopted a real-world comparative effectiveness design aligned with principles of target trial emulation. This approach is particularly appropriate for evaluating complex health IT interventions whose effects depend on workflow integration, implementation support, and sustained use, rather than short-term controlled exposure alone. Of note, Tool C was initially deployed as a standalone platform requiring manual transfer into the EHR, with subsequent integration improving workflow efficiency over time.

The study was approved by the Mass General Brigham Institutional Review Board with a waiver of informed consent owing to its retrospective design and use of deidentified analytic data.

### Participants

The analytic cohort included 163 of the 224 eligible primary care providers, contributing 59 130 provider-days of observation over the study period. Providers were required to have at least 6 months of continuous EHR activity—derived from Signal data, ^17^ a clinician EHR-usage database provided by our EHR vendor—before potential exposure to allow baseline outcome measurement. Sixty-one providers were excluded because corresponding outcome data were not available in Signal data, resulting in the final analytic sample of 163.

Providers were grouped into ITT exposure groups based on their first adoption of an ambient documentation strategy (Table 1): (A) Tablet-Based, Virtual Human-Assisted Ambient Tool (*n* = 65); (B) EHR-Integrated Ambient Tool (*n* = 68); (C) Standalone Ambient Tool (*n* = 17).

An additional 13 providers never adopted an ambient tool during the study period. These were included descriptively in Table 2 but excluded from the main comparative effectiveness analyses, consistent with our design decision to use the EHR-Integrated Ambient Tool as the reference group for vendor comparisons.

**Table 2.** Baseline characteristics of primary care providers by ambient documentation tool.

Tool	None	A	B	C	*P*
		tablet-based, human-assisted ambient tool	integrated EHR ambient tool	standalone/multilingual ambient tool	
**N**	13	65	68	17	
**Sex, male, *N* (%)**	8 (61.5)	18 (27.7)	30 (44.1)	5 (29.4)	.054
**Ethnicity, *N* (%)**					.512
** Asian**	3 (23.1)	16 (24.6)	15 (22.1)	4 (23.5)	
** Black or African American**	1 (7.7)	3 (4.6)	1 (1.5)	2 (11.8)	
** Hispanic or Latino**	1 (7.7)	1 (1.5)	4 (5.9)	1 (5.9)	
** White**	8 (61.5)	40 (61.5)	47 (69.1)	10 (58.8)	
** Missing**	0 (0.0)	5 (7.7)	1 (1.5)	0 (0.0)	
**Age, mean (SD)**	49.46 (15.15)	50.03 (11.29)	51.24 (13.00)	49.12 (10.16)	.888
**Panel size, mean number of patients (SD)**	427.77 (402.28)	730.37 (316.97)	579.41 (285.88)	700.71 (459.49)	.005
**Average patient risk score, mean (SD)**	1.06 (0.41)	1.16 (0.19)	0.88 (0.17)	1.19 (0.34)	<.001
**Switches^a^, mean (SD)**	NaN (NA)	0.35 (0.48)	0.00 (0.00)	0.18 (0.39)	<.001
**Time to first exposure, mean days (SD)**	NaN (NA)	134.40 (46.94)	369.93 (82.95)	175.41 (161.95)	<.001
**Outcome measures during Baseline Period**					
** Pajama time (hours), baseline mean (SD)**	0.12 (0.10)	0.15 (0.14)	0.13 (0.24)	0.09 (0.10)	.458
** Manual composition, mean (SD)**	0.18 (0.17)	0.21 (0.13)	0.13 (0.10)	0.12 (0.13)	.049
** Opportunity score, mean (SD)**	1.14 (0.26)	1.07 (0.32)	1.14 (0.29)	1.06 (0.15)	.749

a Switches reflect the number of providers who switched from one platform to another during the study period.

Values are presented as number (%) unless otherwise indicated. Comparisons are across provider groups (none, Tablet-Based, Human-Assisted Ambient Tool (Tool A), Integrated EHR Ambient Tool (Tool B), Standalone/Multilingual Ambient Tool (Tool C). *P* values are from chi-square or ANOVA tests as appropriate.

### Exposure definition

The primary exposure was adoption of an ambient documentation strategy, defined by the first day of recorded use (the index date). Use of an ambient documentation tool was defined based on EHR-derived metadata capturing tool-generated documentation activity in system logs. The index date corresponded to the first day of recorded use. No prior use was observed before the study window.

For ITT analyses, providers were considered exposed starting at their index date and remained classified in their exposure group thereafter, regardless of discontinuation or switching.

For PP analyses, exposure was defined dynamically: only days during which a provider had documented active use of the assigned strategy were counted as exposed. Non-exposed days for these providers were excluded.

This design intentionally reflects real-world platform switching and phased rollouts, which are intrinsic to enterprise EHR technology adoption and therefore central to understanding pragmatic effectiveness.

### Outcomes

We evaluated 4 prespecified outcomes at the provider-day level, all derived from standardized EHR system logs: Pajama time: Minutes of after-hours EHR activity (5 pm-8 am) per provider-day; Manual composition**:** Proportion of daily note content entered manually by typing or dictation, as opposed to structured templates or ambient tools; Visit closure within 2 days: Binary indicator for whether each encounter was signed within 48 hours, aggregated as a proportion of encounters per provider-day; Opportunity score**:** An EHR-developed composite metric reflecting adoption of efficiency-enhancing EHR functions (eg, SmartPhrases and reuse features), scaled continuously between 0 and 1.

For crude descriptive summaries (Table S2), we reported mean baseline (pre-exposure) and post-exposure values by vendor group, as well as crude deltas. For adjusted analyses, each outcome was modeled separately across multiple follow-up windows.

### Covariates

Covariates included: Provider panel size (average number of active patients during baseline); Panel risk score (mean EHR-generated risk score for the active panel, standardized); Calendar month fixed effects, to account for secular and seasonal trends; Baseline outcome levels, calculated during the 365-day pre-exposure period.^18^ The panel risk score is an EHR-derived composite measure reflecting patient complexity and expected healthcare utilization. Provider age, sex, and ethnicity were described in Table 1 but not included as covariates in adjusted models to avoid overfitting, given collinearity with panel risk score and panel size.

### Statistical analysis

We estimated associations between ambient documentation strategy and outcomes using linear models with an identity link (ordinary least squares applied to bounded outcomes) for proportions (manual composition, visit closure and opportunity score) and linear regression for pajama time. All models incorporated: ITT and PP specifications, month fixed effects, and provider-clustered robust SEs to account for repeated measures. Comparisons were made between Tablet-Based, Virtual Human-Assisted Ambient Tool vs EHR-Integrated Ambient Tool and Standalone Ambient Tool vs EHR-Integrated Ambient Tool, with the EHR-Integrated Ambient Tool serving as the reference vendor throughout.

Our analytical approach was selected to provide interpretable, policy-relevant effect estimates (risk differences) at the provider-day level. Although alternative specifications such as logistic or binomial models can be used for bounded outcomes, these yield odds ratios that are less directly interpretable for operational decision-making. All analyses were performed using R (version 4.4.1) with the fixest package for fixed-effects regression, model summary for tabular presentation, and ggplot2 for visualization.

#### Sensitivity analysis

We repeated analyses separately for 3-, 6-, 9-, and 12-month windows following exposure initiation. To ensure comparability across study windows, each model restricted follow-up to the corresponding time frame.

#### Reporting

This study adhered to the STROBE reporting guidelines for observational studies, and analytic choices were guided by established principles for pragmatic comparative effectiveness evaluation of health IT interventions (Table S1 and Supplementary Text).

## Results

### Study population

A total of 163 primary care providers contributed to the analytic cohort, representing 59 130 provider-days of observation between January 2024 and June 2025. Among these, 150 providers initiated at least 1 ambient documentation strategy during the study period. Of the exposed providers, 65 adopted Tool A [first use/onboarding from March-November 2024], 68 adopted Tool B [first use/onboarding from January 2024 to January 2025], and 17 adopted Tool C [first use/onboarding from January 2024 to January 2025]. Although the number of providers adopting the Standalone Ambient Tool was smaller (*n* = 17), the analytic dataset comprised 59 130 provider-days, providing sufficient statistical precision for estimation, as reflected by the relatively tight 95% CIs reported for most outcomes. Providers included physicians across family medicine, internal medicine, and combined primary care practices (Table 2).

### Baseline characteristics

Table 1 presents provider-level baseline characteristics, measured during the 365-day pre-exposure period. Pajama time: Providers who subsequently adopted Tablet-Based, Virtual Human-Assisted Ambient Tool had the highest pajama time at baseline, averaging more minutes of after-hours EHR use compared with both unexposed providers and those who later adopted Standalone Ambient Tool. By contrast, the EHR-Integrated Ambient Tool adopters demonstrated pajama time levels more similar to those of unexposed providers.

#### Manual composition

All groups demonstrated relatively high baseline manual composition, consistent with limited ambient documentation availability prior to exposure. Visit closure within 2 days: Baseline proportions were similar across groups, with modestly higher closure rates among unexposed providers compared with Standalone Ambient Tool adopters. Opportunity score: This composite indicator was low across all groups at baseline, with only minor differences between modalities. Table S2 provides unadjusted descriptive comparisons of outcomes, stratified by exposure group.

### Intention-to-treat analyses

**Figure 1.**
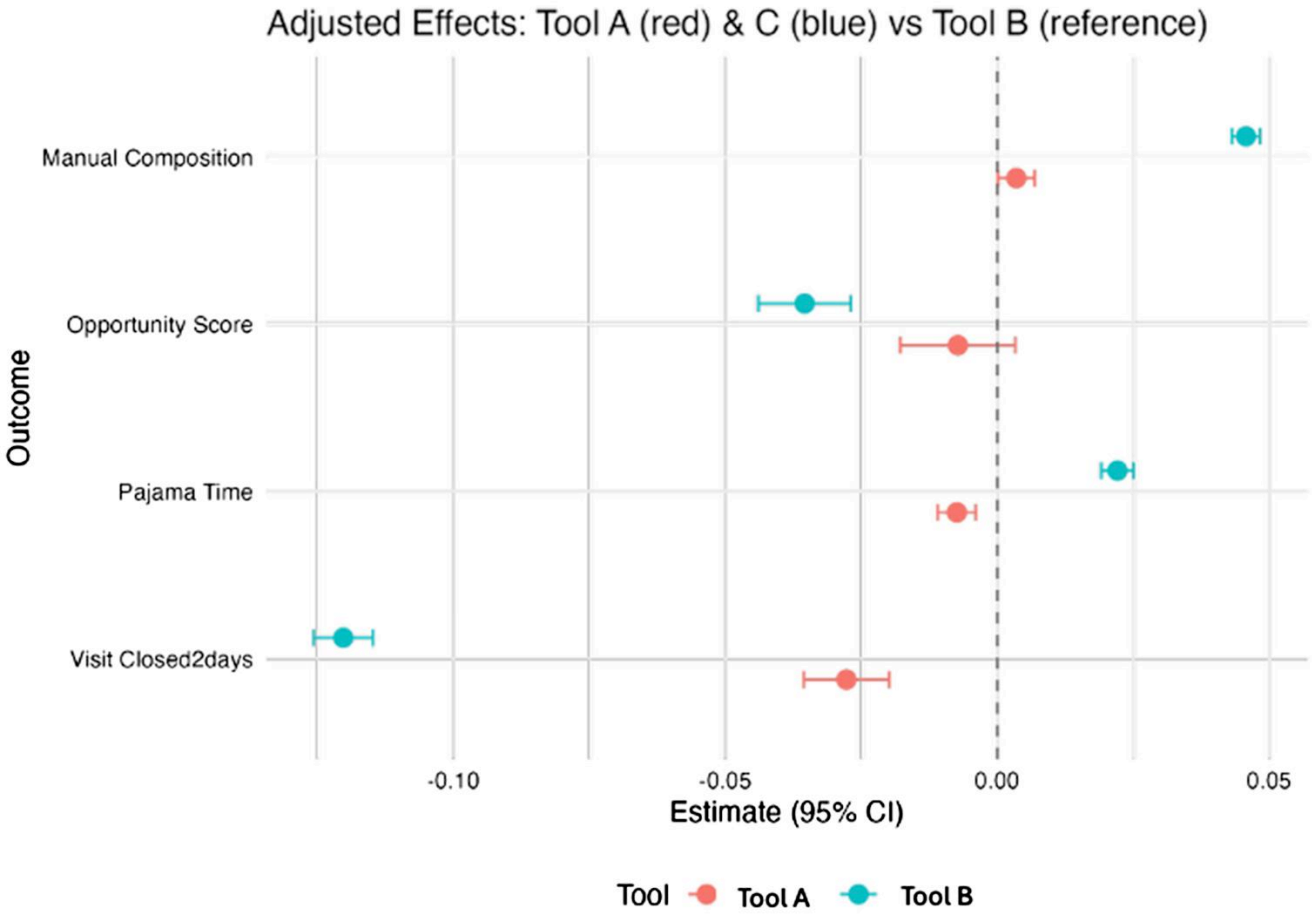
Adjusted effects: Tool A (blue) and C (red) vs Tool B (reference). Tool A (blue) = Tablet-Based, Human-Assisted Ambient Tool. Tool B (reference) = Integrated EHR Ambient Tool. Tool C (red) = Standalone/Multilingual Ambient Tool

In the ITT framework comparing ambient documentation tools with EHR-Integrated Ambient Tool (reference group), significant differences were observed across all outcomes (Figure 1, Tables 2 and 3, Table S3). Providers using Tablet-Based, Virtual Human-Assisted Ambient Tool experienced a substantial increase in pajama time compared with EHR-Integrated Ambient Tool users, with an adjusted marginal effect of +0.022 (95% CI, 0.019-0.025, *P* < .001). In contrast, Standalone Ambient Tool users showed a small but significant decrease in pajama time (−0.007 [−0.011, −0.004], *P* < .001). Regarding manual note composition, both Tablet-Based, Virtual Human-Assisted Ambient Tool and Standalone Ambient Tool were associated with increases relative to EHR-Integrated Ambient Tool, with a larger effect for Tablet-Based, Virtual Human-Assisted Ambient Tool (+0.046 [0.043, 0.048], *P* < .001) and a modest increase for Standalone Ambient Tool (+0.003 [0.000, 0.007], *P* = .042).

**Table 3.** Summary of adjusted effects by tool.

Outcome	Tool	Direction	Interpretation^a^
**Pajama time**	A	↑	Tool A vs Tool B: 0.022 [0.019, 0.025], *P* < .001
**Pajama time**	C	↓	Tool C vs Tool B: −0.007 [−0.011, −0.004], *P* < .001
**Manual composition**	A	↑	Tool A vs Tool B: 0.046 [0.043, 0.048], *P* < .001
**Manual composition**	C	↑	Tool C vs Tool B: 0.003 [0, 0.007], *P* = .042
**Visit closed 2 days**	A	↓	Tool A vs Tool B: −0.12 [−0.126, −0.115], *P* < .001
**Visit closed 2 days**	C	↓	Tool C vs Tool B: −0.028 [−0.036, −0.02], *P* < .001
**Opportunity score**	A	↓	Tool A vs Tool B: −0.035 [−0.044, −0.027], *P* < .001
**Opportunity score**	C	Not significant	Tool C vs Tool B: −0.007 [−0.018, 0.003], *P* = .179

a Adjusted estimates represent differences between each tool and the reference tool (Tool B) from multivariable provider-day–level models with provider-clustered SEs and month fixed effects. Pajama time is measured in hours of after-hours EHR activity (5 pm-8 am) per provider-day; positive values indicate increased after-hours work. Manual composition reflects the proportion (0-1) of daily note content entered manually (typing or dictation); estimates represent absolute percentage-point differences. Visit closure within 2 days is expressed as the proportion (0-1) of encounters signed within 48 hours; negative values indicate lower timely closure rates. Opportunity score is a continuous composite efficiency metric scaled from 0 to 1, with higher values indicating greater use of efficiency-enhancing EHR functions. Direction arrows denote the direction of effect relative to Tool B. Bracketed values indicate 95% CIs; *P* values are 2-sided.

Tool A = Tablet-Based, Human-Assisted Ambient Tool. Tool B = Integrated EHR Ambient Tool. Tool C = Standalone/Multilingual Ambient Tool.

Conversely, visit closure within 2 days—a proxy for timeliness of documentation—was significantly lower among users of both tools compared to EHR-Integrated Ambient Tool, but particularly for Tablet-Based, Virtual Human-Assisted Ambient Tool (−0.120 [−0.126, −0.115], *P* < .001), followed by Standalone Ambient Tool (−0.028 [−0.036, −0.020], *P* < .001). Opportunity scores, representing quality-enhancing opportunities during documentation, were also significantly reduced among Tablet-Based, Virtual Human-Assisted Ambient Tool users (−0.035 [−0.044, −0.027], *P* < .001), while the effect for Standalone Ambient Tool was not significant (−0.007 [−0.018, 0.003], *P* = .179).

These effect sizes correspond to operationally meaningful differences in after-hours workload, documentation timeliness, and workflow efficiency that are directly relevant for health system leaders evaluating platform consolidation, contract renewal, and vendor selection decisions.

### Secondary and sensitivity analyses

PP analyses, restricted to provider-days with documented tool use, yielded effect estimates that were directionally consistent with the primary ITT analysis but generally larger in magnitude. For example, increases in pajama time associated with Tool A were more pronounced under the PP specification, reflecting stronger effects during periods of active tool utilization. Similarly, reductions in visit closure within 2 days were more substantial for both Tool A and Tool C under PP analysis, suggesting that ITT estimates were attenuated by inclusion of days without tool use. Across outcomes, Tool C continued to demonstrate the largest and most consistent effects (Table S4).

Sensitivity analyses restricting follow-up to 3-, 6-, 9-, and 12-month windows demonstrated consistent effect patterns across all time frames (Table S5).

## Discussion

In this large comparative effectiveness study of ambient documentation tools in primary care, we found that adoption of such technologies was associated with substantial, tool-specific differences in workflow efficiency, documentation burden, and after-hours work. These findings demonstrate that ambient documentation platforms are not interchangeable and that tool choice materially shapes real-world outcomes.

Our findings suggest that the EHR-Integrated Ambient Tool may offer more favorable performance across a range of outcomes, while the Tablet-Based, Virtual Human-Assisted Ambient Tool is associated with tradeoffs—improvements in manual documentation possibly at the cost of efficiency—whereas the Standalone Ambient Tool offers de minimis benefits in after-hours work without significant detriment to the opportunity score or reliance on manual note composition.

Recent studies have begun to evaluate ambient documentation tools in real-world clinical settings, with several reports demonstrating improvements in documentation burden, clinician experience, and workflow efficiency.^19–21^ However, most prior work has focused on single tools or single implementations, often relying on self-reported outcomes or uncontrolled designs.^20^^,^^21^ Emerging evidence from multisite and system-level evaluations has reinforced the potential for ambient technologies to reduce documentation time and improve usability, while also highlighting variability in performance depending on implementation context, workflow integration, and tool design.^19–21^ Our findings are consistent with this broader literature in demonstrating measurable improvements in documentation-related outcomes, while extending it by providing direct, head-to-head comparisons across multiple tools within the same health system.^12^^,^^22–25^

This study contributes to the literature in several important ways. First, it represents one of the first comparative evaluations of multiple ambient documentation tools within a single health system, allowing a direct assessment of tool-specific differences under similar clinical and operational conditions. Second, it leverages EHR-derived, daily provider-level measures to objectively quantify outcomes such as pajama time, manual composition, and timely visit closure, avoiding reliance on self-reported metrics. Third, it applies a consistent analytic framework across tools and outcomes, enabling robust comparisons of relative effectiveness. Together, these features provide pragmatic, real-world evidence that is directly relevant to health system leaders making decisions about technology adoption, optimization, and consolidation.

Notably, our findings differ in part from prior reports by suggesting that the standalone ambient tool may not consistently outperform EHR-integrated approaches across all operational metrics.^19–21^ While some recent studies have reported strong performance of more flexible or AI-driven tools, differences in study design, implementation maturity, and workflow integration likely contribute to variation in observed effects.^19–21^ In particular, our results suggest that integration within the EHR and alignment with existing clinical workflows may play a more central role in driving consistent improvements than previously appreciated.

Several mechanisms may explain the tool-specific differences observed. Workflow integration: The Standalone Ambient Tool may benefit from lightweight deployment and adaptability once integrated into the EHR, although its smaller user base limits precision. Tablet-Based, Virtual Human-Assisted Ambient Tool, by contrast, requires more rigid workflows, slowing adoption and reducing day-to-day efficiency gains.

Speech recognition, natural language processing quality, and large language model performance: Differences in model training, domain-specific tuning, and error handling likely contribute. Higher transcription fidelity and better contextual understanding may reduce manual editing needs, thereby lowering pajama time and manual composition.

Support and implementation infrastructure: Differences in rollout strategies, including variation in EHR integration, workflow design, degree of automation, and implementation support (eg, training, troubleshooting, and ongoing clinician feedback), may influence adoption and sustained benefit of ambient documentation tools (Table 1). For example, tools requiring manual transfer or additional workflow steps may introduce early friction, whereas more integrated and automated approaches may facilitate faster uptake. These implementation and design differences likely contribute to observed variation in effectiveness and underscore the importance of aligning tool selection and deployment strategies with clinical workflows.

Clinician selection and baseline burden: Baseline imbalances were observed across groups, with Tablet-Based, Virtual Human-Assisted Ambient Tool adopters demonstrating higher pajama time prior to adoption. While our time-varying models naturally adjust for baseline outcomes, residual confounding cannot be excluded. Nevertheless, our findings highlight that not all tools deliver equivalent benefits, emphasizing the importance of vendor selection and evidence-based procurement in digital health adoption.

This study has several notable strengths. Large, diverse sample: The analytic cohort comprised primary care providers across multiple practice sites, including physicians and APCs. This diversity enhances generalizability to real-world primary care.

Objective, EHR-derived outcomes: Unlike prior studies relying on surveys or self-report, our analysis used daily provider-level measures directly extracted from the EHR, including pajama time, note closure, and manual composition.^11^^,^^12^^,^^22^^,^^24^^,^^26^ These outcomes provide a reliable, high-resolution view of documentation burden and efficiency.

Comparative, platform-specific design: By examining multiple ambient documentation tools, with different characteristics, within the same health system, we were able to generate head-to-head evidence on platform performance while holding many contextual factors constant.

Robust analytic framework: The combination of ITT, PP, and sensitivity analyses allowed us to account for variable adoption patterns, secular trends, and unmeasured biases. Clustering by provider and inclusion of month fixed effects further strengthened the approach.

Several limitations should be considered when interpreting these findings.

Outcomes such as visit closure were modeled as provider-day proportions using linear models. Although alternative approaches (eg, logistic or binomial models) could be applied, our primary goal was to estimate absolute differences in performance, which are more directly interpretable for health system decision-making. Additionally, encounter-level numerator and denominator data were not available in the analytic dataset, precluding binomial modeling.

Non-randomized design: As with all observational studies, residual confounding remains possible. Providers who selected a particular tool may differ in motivation, prior experience with scribing, workflow preferences, or patient mix. Some clinicians—such as certified bilingual staff caring for large numbers of non-English-speaking patients—reportedly transitioned from one modality to another early in the study period because of support for multilingual language-concordant visits. Such platform switching, as well as prior experience with one system, could influence subsequent engagement with and use of another platform, introducing additional potential for unmeasured confounding.^27^^,^^28^ However, for rapidly deployed enterprise health IT interventions, observational comparative effectiveness designs are uniquely capable of capturing pragmatic workflow integration effects, sustained adoption patterns, and implementation heterogeneity that randomized trials cannot readily reproduce.

Baseline imbalances: Although we adjusted for pre-adoption outcomes, Tablet-Based, Virtual Human-Assisted Ambient Tool adopters had higher pajama time at baseline. This may have increased the opportunity for improvement. We also did not capture provider-level qualified bilingual status or detailed patient panel composition (eg, language or payer mix), which could contribute to unmeasured confounding. Nonetheless, the magnitude and persistence of the observed effects suggest true differences in tool effectiveness.

Single regional health system: Findings may not generalizable beyond a large academic system with robust informatics infrastructure. Smaller practices or different organizational contexts may experience different results.

Measurement limitation for Standalone Ambient Tool: Prior to EHR integration, providers may have edited notes directly within the standalone portal; this activity is not captured in our EHR-based time metrics, and the vendor does not provide time-stamped activity data (eg, time on site or active editing duration). Consequently, we cannot fully account for pre-integration editing time and can evaluate usage patterns with fidelity only after integration became available; pre-integration efficiency may therefore be underestimated.

Limited sample size for Standalone Ambient Tool: Although patterns were consistent, estimates for the Standalone Ambient Tool were less precise due to smaller provider numbers. Nevertheless, inference is supported by the large number of provider-days and relatively tight 95% CIs for the primary outcomes. These findings should be interpreted as preliminary and hypothesis generating pending confirmation in larger, multisystem cohorts.

Outcome scope: We focused on efficiency, burden, and productivity metrics. Other important outcomes—such as note quality, patient experience, or downstream clinical outcomes—were not directly assessed.

## Conclusion

Ambient documentation tools are increasingly deployed to reduce documentation burden and help alleviate clinician burnout, but our findings demonstrate that tool choice materially shapes real-world outcomes.^10^^,^^24^ Our findings confirm that adoption can meaningfully improve efficiency, but they also show that differences in tool attributes lead to divergent outcomes. Without careful evaluation, health systems risk introducing new sources of burden, error, or workflow disruption—the opposite of what these technologies are intended to achieve.

Health systems must therefore recognize that workflow and implementation approaches matters; investments should be guided by rigorous comparative evidence rather than marketing claims. Policymakers and payers should consider supporting the adoption of tools with demonstrated effectiveness, as efficiency gains can translate into improved provider retention, enhanced patient experience, and long-term value for care delivery. This type of evidence is especially critical given the rapid pace of technological evolution in this domain.^6–9^

Future research should expand beyond efficiency outcomes to assess note quality, patient safety, clinical communication, and financial sustainability. Randomized or quasi-experimental designs (eg, stepped-wedge trials) could strengthen causal inference, ^27^^,^^28^ while qualitative work exploring provider experiences after switching tools may help clarify mechanisms driving tool-specific differences.

Emerging advances in large language models and generative AI may further transform ambient documentation.^6–9^ Studies should evaluate whether these next-generation tools achieve greater efficiency gains, improved accuracy, seamless integration, and reduced unintended consequences compared with current offerings.

In conclusion, while ambient documentation technologies hold real promise for addressing documentation burden and clinician burnout, our results demonstrate that ongoing, rigorous evaluation is essential to ensure they deliver on their intended goals without unintended harm.

## Supplementary Material

ooag155_Supplementary_Data

## Data Availability

De-identified data underlying this study may be made available upon reasonable request, subject to institutional review and execution of a Data Use Agreement (DUA) in accordance with applicable regulations and policies.
