# Supplementary material for: Comparative effectiveness of ambient documentation tools in primary care tool-specific variations in efficiency, documentation burden, and productivity over time
Source: JAMIA Open. 2026 Aug 11;9(4):ooag155. doi: 10.1093/jamiaopen/ooag155 (PMC13458937; doi:10.1093/jamiaopen/ooag155)
Supplement: Supplementary file 1 [file ooag155_supplementary_data.docx]

**Supplementary Figure 1**

**Supplementary Table 1 – STROBE Checklist for Reporting Observational Studies**

| **Checklist Item** | **Recommendation** | **Location in Manuscript** |
| --- | --- | --- |
| Title and abstract | Indicate the study's design with a commonly used term in the title or the abstract; provide an informative and balanced summary. | Page 1-3 |
| Background/rationale | Explain the scientific background and rationale for the investigation being reported. | Page 5 |
| Objectives | State specific objectives, including any prespecified hypotheses. | Page 2 |
| Study design | Present key elements of study design early in the paper. | Page 6 |
| Setting | Describe the setting, locations, and relevant dates, including periods of recruitment, exposure, follow-up, and data collection. | Page 6 |
| Participants | Give eligibility criteria, and the sources and methods of selection of participants. Describe methods of follow-up. | Page 6 |
| Variables | Clearly define all outcomes, exposures, predictors, potential confounders, and effect modifiers. Give diagnostic criteria, if applicable. | Page 7-8 |
| Data sources/measurement | For each variable of interest, give sources of data and details of methods of assessment (measurement). | Page 8-9 |
| Bias | Describe any efforts to address potential sources of bias. | Page 7, 11-12 |
| Study size | Explain how the study size was arrived at. | Page 8 |
| Quantitative variables | Explain how quantitative variables were handled in the analyses. If applicable, describe which groupings were chosen and why. | Page 6-8 |
| Statistical methods | Describe all statistical methods, including those used to control for confounding. Describe any methods used to examine subgroups and interactions. Explain how missing data were addressed. | Page 7-8 |
| Participants (Results) | Report numbers of individuals at each stage of study—e.g., numbers potentially eligible, examined for eligibility, confirmed eligible, included in the study, completing follow-up, and analysed. | Page 8 |
| Descriptive data | Give characteristics of study participants (e.g., demographic, clinical, social) and information on exposures and potential confounders. | Page 8 |
| Outcome data | Report numbers of outcome events or summary measures over time. | Page 8-9 |
| Main results | Give unadjusted estimates and, if applicable, confounder-adjusted estimates and their precision (e.g., 95% confidence interval). | Page 8-9 |
| Other analyses | Report other analyses done—e.g., analyses of subgroups and interactions, and sensitivity analyses. | Page 9 |
| Key results | Summarize key results with reference to study objectives. | Page 8-9 |
| Limitations | Discuss limitations of the study, considering sources of potential bias or imprecision. Discuss both direction and magnitude of any potential bias. | Page 11-12 |
| Interpretation | Give a cautious overall interpretation of results considering objectives, limitations, multiplicity of analyses, results from similar studies, and other relevant evidence. | Page 10-12 |
| Generalizability | Discuss the generalizability (external validity) of the study results. | Page 11 |
| Funding | Give the source of funding and the role of the funders for the present study and, if applicable, for the original study on which the present article is based. | Page 12 |

**Supplementary Table 2 – Crude Pre- and Post-Exposure Outcome Measures by Ambient Documentation Tool**

| **exposure_group** | **outcome** | **n_providers** | **mean_pre** | **mean_post** | **delta** |
| --- | --- | --- | --- | --- | --- |
| Tool A | opportunity_score_time_varying | 65 | 0.0199716759445443 | 0.862509198065717 | 0.842537522121173 |
| Tool A | p_manual_composition_time_varying | 65 | 0.00217809351380968 | 0.177041261750767 | 0.174863168236957 |
| Tool A | p_pajama_time_time_varying | 65 | 0.00263701399695806 | 0.123775317316704 | 0.121138303319746 |
| Tool A | p_visit_closed2days_time_varying | 65 | 0.0151565684337765 | 0.573010446103369 | 0.557853877669592 |
| Tool B | opportunity_score_time_varying | 68 | 0.603555047502557 | 1.02545468738245 | 0.421899639879892 |
| Tool B | p_manual_composition_time_varying | 68 | 0.0925832692786544 | 0.161922203836946 | 0.0693389345582919 |
| Tool B | p_pajama_time_time_varying | 68 | 0.0475990814952355 | 0.0753434429496198 | 0.0277443614543842 |
| Tool B | p_visit_closed2days_time_varying | 68 | 0.491252205586109 | 0.88743918267003 | 0.396186977083921 |
| Tool C | opportunity_score_time_varying | 16 | 0.266615850237683 | 0.826759284721795 | 0.560143434484111 |
| Tool C | p_manual_composition_time_varying | 16 | 0.0448815771858576 | 0.109914928141144 | 0.0650333509552863 |
| Tool C | p_pajama_time_time_varying | 16 | 0.0169060411117837 | 0.0723260359880027 | 0.055419994876219 |
| Tool C | p_visit_closed2days_time_varying | 16 | 0.204828231940042 | 0.644518320592648 | 0.439690088652607 |

Legend: Values represent means during baseline (pre) and follow-up (post) periods. Delta represents the crude change (post − pre).

**Supplementary Table 3 – All model results**

| **term** | **estimate** | **std.error** | **statistic** | **p.value** | **conf.low** | **conf.high** | **model** | **outcome** |
| --- | --- | --- | --- | --- | --- | --- | --- | --- |
| **(Intercept)** | 0.05361480507277850 | 6.80725472173401E-04 | 6203.338198832340 | 0 | 0.05228060766395960 | 0.05494900248159730 | crude | p_pajama_time_time_varying |
| **exposure_groupsToolA** | 0.033235229672228500 | 0.001095332490886570 | 920.6730442370890 | 0 | 0.031088417438994300 | 0.035382041905462700 | crude | p_pajama_time_time_varying |
| **exposure_groupToolC** | 0.006011752321256650 | 0.0014315205279961100 | 17.63628056437530 | 2.67437481513699E-05 | 0.003206023643254510 | 0.0088174809992588 | crude | p_pajama_time_time_varying |
| **(Intercept)** | 0.0685942959370072 | 9.96005367922606E-04 | 4742.9947889745600 | 0 | 0.06664216128747030 | 0.07054643058654410 | volume | p_pajama_time_time_varying |
| **exposure_groupToolA** | 0.03713792223309120 | 0.0011285388765701600 | 1082.9344159849300 | 0 | 0.034926026679860400 | 0.03934981778632210 | volume | p_pajama_time_time_varying |
| **exposure_groupToolC** | 0.010221597558318000 | 0.0014376389597477400 | 50.551947225425500 | 1.16051612764068E-12 | 0.007403876974440760 | 0.013039318142195200 | volume | p_pajama_time_time_varying |
| **n_patients** | -2.58529283951367E-05 | 1.11026564675186E-06 | 542.2077015296770 | 0 | -2.80290090760425E-05 | -2.3676847714231E-05 | volume | p_pajama_time_time_varying |
| **(Intercept)** | 0.0034105440829336100 | 0.0025780958094181400 | 1.7500450513315900 | 0.18587106888660400 | -0.0016424308522195900 | 0.008463519018086800 | risk | p_pajama_time_time_varying |
| **exposure_groupToolA** | 0.01747205114169460 | 0.001539551685278470 | 128.79506942447300 | 0 | 0.014454585286210800 | 0.020489516997178300 | risk | p_pajama_time_time_varying |
| **exposure_groupToolC** | -0.012584287909385600 | 0.00187173548820868 | 45.203112763700100 | 1.7762236126373E-11 | -0.01625282205486010 | -0.008915753763911100 | risk | p_pajama_time_time_varying |
| **average_patient_risk_score** | 0.05672850491861340 | 0.003189694512811610 | 316.30386097043100 | 0 | 0.050476818551817600 | 0.06298019128540920 | risk | p_pajama_time_time_varying |
| **(Intercept)** | 0.02000091462960960 | 0.0029056440855325300 | 47.38216272886850 | 5.84121639946034E-12 | 0.014305956870074100 | 0.025695872389145200 | full | p_pajama_time_time_varying |
| **exposure_groupToolA** | 0.02208563007081380 | 0.00162847490047284 | 183.93206584955800 | 0 | 0.018893877916159600 | 0.025277382225468000 | full | p_pajama_time_time_varying |
| **exposure_groupToolC** | -0.007434806153227550 | 0.0019302487968284300 | 14.835859731648700 | 1.17284077100988E-04 | -0.011218024276213000 | -0.003651588030242050 | full | p_pajama_time_time_varying |
| **n_patients** | -1.922966643408E-05 | 1.14901419709648E-06 | 280.0868453337390 | 0 | -2.14816928781143E-05 | -1.69776399900457E-05 | full | p_pajama_time_time_varying |
| **average_patient_risk_score** | 0.05057197820479130 | 0.0032631575414587800 | 240.18384951264900 | 0 | 0.044176306947651800 | 0.056967649461930700 | full | p_pajama_time_time_varying |
| **(Intercept)** | -0.08567310570416170 | 0.0030585306239324200 | 784.6271915639450 | 0 | -0.091667715572682100 | -0.07967849583564140 | spline30 | p_pajama_time_time_varying |
| **exposure_groupToolA** | 0.022085630070811200 | 0.001513789894264290 | 212.85721902782500 | 0 | 0.019118656397892400 | 0.025052603743729900 | spline30 | p_pajama_time_time_varying |
| **exposure_groupToolC** | -0.007434806153230520 | 0.0017977668891479700 | 17.103009798616900 | 3.54061502574332E-05 | -0.010958364508559200 | -0.003911247797901890 | spline30 | p_pajama_time_time_varying |
| **n_patients** | -1.92296664340644E-05 | 1.06805303353925E-06 | 324.15883731827800 | 0 | -2.13230119133801E-05 | -1.71363209547487E-05 | spline30 | p_pajama_time_time_varying |
| **average_patient_risk_score** | 0.05057197820480220 | 0.0030621069559323700 | 272.75905185260000 | 0 | 0.04457035885436520 | 0.056573597555239300 | spline30 | p_pajama_time_time_varying |
| **ns(time_days, knots = 30)1** | 0.20742726530750700 | 0.0025138942453300900 | 6808.284191861030 | 0 | 0.20250012312571700 | 0.21235440748929600 | spline30 | p_pajama_time_time_varying |
| **ns(time_days, knots = 30)2** | 0.14481483689175700 | 0.001525243249643050 | 9014.629785060300 | 0 | 0.14182541505479400 | 0.14780425872872000 | spline30 | p_pajama_time_time_varying |
| **(Intercept)** | 0.0974181638197278 | 8.74253568383263E-04 | 12416.667509605000 | 0 | 0.09570465831234100 | 0.09913166932711460 | crude | p_manual_composition_time_varying |
| **exposure_groupToolA** | 0.02864561524791950 | 0.0013265349519776700 | 466.3144935964050 | 0 | 0.026045654517809700 | 0.031245575978029300 | crude | p_manual_composition_time_varying |
| **exposure_groupToolC** | -0.016430857663549700 | 0.0017955779749828300 | 83.73594535937360 | 0 | -0.01995012582594940 | -0.01291158950115000 | crude | p_manual_composition_time_varying |
| **(Intercept)** | 0.10162986165510800 | 0.0016011495817920700 | 4028.829201548920 | 0 | 0.09849166614093440 | 0.10476805716928200 | volume | p_manual_composition_time_varying |
| **exposure_groupToolA** | 0.02974291301057690 | 0.0013594919381887700 | 478.6452228047240 | 0 | 0.027078357774454400 | 0.03240746824669950 | volume | p_manual_composition_time_varying |
| **exposure_groupToolC** | -0.015247199538373100 | 0.0017380222429937500 | 76.9607882871484 | 0 | -0.01865366053897040 | -0.01184073853777590 | volume | p_manual_composition_time_varying |
| **n_patients** | -7.26892012200209E-06 | 2.2666157080148E-06 | 10.28451946579030 | 0.0013415095239218700 | -1.17114052765039E-05 | -2.82643496750033E-06 | volume | p_manual_composition_time_varying |
| **(Intercept)** | 0.14086542938064900 | 0.0027278094237674900 | 2666.7407417020300 | 0 | 0.13551902115337500 | 0.1462118376079220 | risk | p_manual_composition_time_varying |
| **exposure_groupToolA** | 0.042287226342273500 | 0.0015477513007155100 | 746.4763369611480 | 0 | 0.03925368953584610 | 0.045320763148700900 | risk | p_manual_composition_time_varying |
| **exposure_groupToolC** | -3.37659947764814E-04 | 0.002032638384737980 | 0.027595537187193600 | 0.868063266469332 | -0.004321557975444930 | 0.0036462380799153 | risk | p_manual_composition_time_varying |
| **average_patient_risk_score** | -0.04909341098703330 | 0.002795875811481530 | 308.3263642765650 | 0 | -0.05457322688278380 | -0.04361359509128280 | risk | p_manual_composition_time_varying |
| **(Intercept)** | 0.15320106065548600 | 0.003012221362833870 | 2586.722112423460 | 0 | 0.14729721527086900 | 0.15910490604010200 | full | p_manual_composition_time_varying |
| **exposure_groupToolA** | 0.04571761437169380 | 0.0015656178568956700 | 852.698674522505 | 0 | 0.04264905975862540 | 0.048786168984762100 | full | p_manual_composition_time_varying |
| **exposure_groupToolC** | 0.003491194146947060 | 0.001943897469423310 | 3.225531539499040 | 0.07249825672186870 | -3.18774882761183E-04 | 0.0073011631766553000 | full | p_manual_composition_time_varying |
| **n_patients** | -1.42980576595882E-05 | 2.21479889440435E-06 | 41.67594844543420 | 1.07726050302404E-10 | -1.86389837256198E-05 | -9.9571315935565E-06 | full | p_manual_composition_time_varying |
| **average_patient_risk_score** | -0.05367104493805550 | 0.0027264885282126400 | 387.50093265757700 | 0 | -0.05901486425761390 | -0.04832722561849710 | full | p_manual_composition_time_varying |
| **(Intercept)** | -0.016082661247592500 | 0.0025036360776609100 | 41.26419969197530 | 1.32982291844996E-10 | -0.020989697790203000 | -0.011175624704982000 | spline30 | p_manual_composition_time_varying |
| **exposure_groupToolA** | 0.04571761437170810 | 0.001316036595981650 | 1206.788568793470 | 0 | 0.0431382300412474 | 0.04829699870216880 | spline30 | p_manual_composition_time_varying |
| **exposure_groupToolC** | 0.0034911941469654200 | 0.0017191464734228000 | 4.124034029654540 | 0.04227815079361770 | 1.21728974907689E-04 | 0.006860659319023160 | spline30 | p_manual_composition_time_varying |
| **n_patients** | -1.42980576595855E-05 | 1.89650220626428E-06 | 56.83912500541840 | 4.72955008490317E-14 | -1.80151336804642E-05 | -1.05809816387067E-05 | spline30 | p_manual_composition_time_varying |
| **average_patient_risk_score** | -0.05367104493810910 | 0.002258773588597940 | 564.5919733625030 | 0 | -0.058098159820991400 | -0.049243930055226900 | spline30 | p_manual_composition_time_varying |
| **ns(time_days, knots = 30)1** | 0.3365123138998070 | 0.0029889701620009100 | 12675.315043373200 | 0 | 0.3306540400314200 | 0.3423705877681930 | spline30 | p_manual_composition_time_varying |
| **ns(time_days, knots = 30)2** | 0.2204451965761200 | 0.0016540463097983700 | 17762.576070175400 | 0 | 0.217203325380154 | 0.22368706777208600 | spline30 | p_manual_composition_time_varying |
| **(Intercept)** | 0.5293380619163490 | 0.0028977903894783800 | 33368.163926840000 | 0 | 0.523658497118225 | 0.5350176267144730 | crude | p_visit_closed2days_time_varying |
| **exposure_groupToolA** | -0.1169597322182800 | 0.003846469563893530 | 924.5877389540270 | 0 | -0.1244986740311410 | -0.1094207904054200 | crude | p_visit_closed2days_time_varying |
| **exposure_groupToolC** | -0.023973017393752700 | 0.0064045180424563800 | 14.011108431085900 | 1.81733806240647E-04 | -0.036525642095304200 | -0.01142039269220120 | crude | p_visit_closed2days_time_varying |
| **(Intercept)** | 0.5266389532471890 | 0.004381328827795400 | 14448.217126335700 | 0 | 0.5180517065402830 | 0.5352261999540950 | volume | p_visit_closed2days_time_varying |
| **exposure_groupToolA** | -0.11766294646093600 | 0.003958586356220500 | 883.4850241393230 | 0 | -0.12542163314882000 | -0.10990425977305200 | volume | p_visit_closed2days_time_varying |
| **exposure_groupToolC** | -0.02473157653818500 | 0.00647284099167147 | 14.59868462170370 | 1.3300735233146E-04 | -0.03741811175951570 | -0.012045041316854400 | volume | p_visit_closed2days_time_varying |
| **n_patients** | 4.65836013957753E-06 | 5.73613882866629E-06 | 0.659518244911565 | 0.4167300724583310 | -6.58426537493017E-06 | 1.59009856540852E-05 | volume | p_visit_closed2days_time_varying |
| **(Intercept)** | 0.5233636052878710 | 0.008583335083234490 | 3717.876014546460 | 0 | 0.506540577657492 | 0.5401866329182490 | risk | p_visit_closed2days_time_varying |
| **exposure_groupToolA** | -0.11883559742577000 | 0.0045637659550778200 | 678.0260488246850 | 0 | -0.1277804143315930 | -0.10989078051994800 | risk | p_visit_closed2days_time_varying |
| **exposure_groupToolC** | -0.026186001583305900 | 0.007111994851941700 | 13.556747662237300 | 2.31457814826364E-04 | -0.04012525535134590 | -0.012246747815265900 | risk | p_visit_closed2days_time_varying |
| **average_patient_risk_score** | 0.006750861093300960 | 0.009122484602514060 | 0.5476360961791210 | 0.4592851957962750 | -0.01112888017714780 | 0.024630602363749700 | risk | p_visit_closed2days_time_varying |
| **(Intercept)** | 0.5183725443033370 | 0.01003468851169490 | 2668.5551947253100 | 0 | 0.4987049162243370 | 0.5380401723823370 | full | p_visit_closed2days_time_varying |
| **exposure_groupToolA** | -0.12022355037878500 | 0.004808394837154540 | 625.1422535869160 | 0 | -0.1296478310830570 | -0.11079926967451400 | full | p_visit_closed2days_time_varying |
| **exposure_groupToolC** | -0.027735175992101100 | 0.007303741426418700 | 14.420192999168900 | 1.46225908689956E-04 | -0.0420502461402749 | -0.013420105843927200 | full | p_visit_closed2days_time_varying |
| **n_patients** | 5.78506897185967E-06 | 5.88154205550996E-06 | 0.9674636774064370 | 0.32531354600609400 | -5.74254163049752E-06 | 1.73126795742169E-05 | full | p_visit_closed2days_time_varying |
| **average_patient_risk_score** | 0.008602995751597230 | 0.009351790741824230 | 0.8462717743547930 | 0.35760917873705900 | -0.00972617729333337 | 0.026932168796527800 | full | p_visit_closed2days_time_varying |
| **(Intercept)** | -0.20920224539129900 | 0.006343824171141040 | 1087.5028616589200 | 0 | -0.2216359122909910 | -0.19676857849160800 | spline30 | p_visit_closed2days_time_varying |
| **exposure_groupToolA** | -0.12022355037873800 | 0.0027701083164698300 | 1883.5866170079500 | 0 | -0.12565286291229400 | -0.11479423784518200 | spline30 | p_visit_closed2days_time_varying |
| **exposure_groupToolC** | -0.027735175992050100 | 0.004003458811370020 | 47.99446142644760 | 4.27424762250439E-12 | -0.03558181107592490 | -0.019888540908175300 | spline30 | p_visit_closed2days_time_varying |
| **n_patients** | 5.78506897181966E-06 | 3.56465883436819E-06 | 2.6337899773688600 | 0.1046117342740100 | -1.20153396071453E-06 | 1.27716719043538E-05 | spline30 | p_visit_closed2days_time_varying |
| **average_patient_risk_score** | 0.008602995751451110 | 0.005360893162030760 | 2.5752838982146200 | 0.10854462621813200 | -0.0019041617710962100 | 0.01911015327399840 | spline30 | p_visit_closed2days_time_varying |
| **ns(time_days, knots = 30)1** | 1.4260264254501600 | 0.007357753812168330 | 37563.3781266904 | 0 | 1.4116054929712000 | 1.4404473579291200 | spline30 | p_visit_closed2days_time_varying |
| **ns(time_days, knots = 30)2** | 1.002874727136940 | 0.003020489378340120 | 110239.88420450600 | 0 | 0.9969546767397090 | 1.0087947775341700 | spline30 | p_visit_closed2days_time_varying |
| **(Intercept)** | 0.6479919918818700 | 0.0037890843725961600 | 29246.28619809690 | 0 | 0.6405655229771980 | 0.6554184607865420 | crude | opportunity_score_time_varying |
| **exposure_groupToolA** | -0.030516298845765300 | 0.005397446715069890 | 31.965904043534500 | 1.5690249655087E-08 | -0.04109510001577640 | -0.01993749767575430 | crude | opportunity_score_time_varying |
| **exposure_groupToolC** | -0.002013083809066690 | 0.00816409416983463 | 0.06080057700218200 | 0.8052350412273560 | -0.018014414348336000 | 0.013988246730202600 | crude | opportunity_score_time_varying |
| **(Intercept)** | 0.627239086052094 | 0.005946050018324900 | 11127.794845612000 | 0 | 0.6155850421659040 | 0.6388931299382850 | volume | opportunity_score_time_varying |
| **exposure_groupToolA** | -0.035923172278911900 | 0.005549494362944480 | 41.90275068724090 | 9.59275991974096E-11 | -0.04679998136269120 | -0.02504636319513270 | volume | opportunity_score_time_varying |
| **exposure_groupToolC** | -0.00784549310467974 | 0.00820588113574347 | 0.9140922912742890 | 0.3390309399424640 | -0.023928724592153600 | 0.008237738382794090 | volume | opportunity_score_time_varying |
| **n_patients** | 3.58171978786175E-05 | 7.84262888903644E-06 | 20.85738620997320 | 4.94780562942943E-06 | 2.04459277119927E-05 | 5.11884680452423E-05 | volume | opportunity_score_time_varying |
| **(Intercept)** | 0.6595849064077500 | 0.01184103351665030 | 3102.8601158661800 | 0 | 0.6363769071753840 | 0.6827929056401170 | risk | opportunity_score_time_varying |
| **exposure_groupToolA** | -0.02687634522901980 | 0.006482340683047400 | 17.190033309788600 | 3.38206094995241E-05 | -0.03958149950331150 | -0.01417119095472810 | risk | opportunity_score_time_varying |
| **exposure_groupToolC** | 0.00228101993171021 | 0.009240601823688120 | 0.06093371352908470 | 0.8050262084051910 | -0.015830226838193600 | 0.020392266701614100 | risk | opportunity_score_time_varying |
| **average_patient_risk_score** | -0.01309946000081170 | 0.012749151742444300 | 1.0557089558615900 | 0.30419591699858700 | -0.03808733824943870 | 0.011888418247815300 | risk | opportunity_score_time_varying |
| **(Intercept)** | 0.628876172878189 | 0.013563033056133900 | 2149.8919827762200 | 0 | 0.6022931165670400 | 0.6554592291893380 | full | opportunity_score_time_varying |
| **exposure_groupToolA** | -0.03541606804917360 | 0.006767428210084940 | 27.387561894855400 | 1.66498706266616E-07 | -0.04867998360890040 | -0.02215215248944670 | full | opportunity_score_time_varying |
| **exposure_groupToolC** | -0.007250657653203680 | 0.009385441868211090 | 0.5968227257023860 | 0.43979287441979500 | -0.025645785693891700 | 0.01114447038748440 | full | opportunity_score_time_varying |
| **n_patients** | 3.5594063480073E-05 | 7.95601381617809E-06 | 20.015391256603800 | 7.6821336887356E-06 | 2.00005629398609E-05 | 5.11875640202851E-05 | full | opportunity_score_time_varying |
| **average_patient_risk_score** | -0.0017037447704305000 | 0.012941327366859500 | 0.01733210952940150 | 0.8952599726932940 | -0.027068280321617600 | 0.023660790780756600 | full | opportunity_score_time_varying |
| **(Intercept)** | -0.34811235534604000 | 0.009368373670913150 | 1380.7354192237800 | 0 | -0.36647403033474300 | -0.329750680357337 | spline30 | opportunity_score_time_varying |
| **exposure_groupToolA** | -0.035416068049316200 | 0.004336530762604350 | 66.69846263608600 | 3.33066907387547E-16 | -0.04391551216187070 | -0.02691662393676160 | spline30 | opportunity_score_time_varying |
| **exposure_groupToolC** | -0.007250657653370720 | 0.0053987080284883000 | 1.8037449100000400 | 0.17926041207504200 | -0.01783193095225500 | 0.0033306156455135900 | spline30 | opportunity_score_time_varying |
| **n_patients** | 3.55940634801499E-05 | 5.20397052934995E-06 | 46.78272170601370 | 7.93098919871227E-12 | 2.53944686660161E-05 | 4.57936582942836E-05 | spline30 | opportunity_score_time_varying |
| **average_patient_risk_score** | -0.001703744769928610 | 0.008244048955704980 | 0.04270984026429690 | 0.8362725928927630 | -0.017861783809895400 | 0.014454294270038200 | spline30 | opportunity_score_time_varying |
| **ns(time_days, knots = 30)1** | 1.920679504994110 | 0.010353926659811000 | 34411.18556072500 | 0 | 1.9003861816423100 | 1.9409728283459100 | spline30 | opportunity_score_time_varying |
| **ns(time_days, knots = 30)2** | 1.3307979618194800 | 0.004752664799783950 | 78406.10574887430 | 0 | 1.3214829099813100 | 1.340113013657650 | spline30 | opportunity_score_time_varying |

**Supplementary Table 4 – Per-protocol**

| **term** | **estimate** | **std.error** | **statistic** | **p.value** | **conf.low** | **conf.high** | **outcome** | **analysis** | **protocol** | **window_months** | **n_rows** | **n_providers** |
| --- | --- | --- | --- | --- | --- | --- | --- | --- | --- | --- | --- | --- |
| **(Intercept)** | -0.08567310570416010 | 0.003058530623933400 | 784.6271915634130 | 0 | -0.09166771557268240 | -0.07967849583563780 | p_pajama_time_time_varying | PerProtocol_actual_use | PP | 12 | 54385 | 149 |
| **exposure_groupabridge** | -0.007434806153230080 | 0.001797766889148230 | 17.103009798609900 | 3.54061502575442E-05 | -0.01095836450855920 | -0.003911247797900940 | p_pajama_time_time_varying | PerProtocol_actual_use | PP | 12 | 54385 | 149 |
| **exposure_groupscribe** | 0.022085630070811500 | 0.0015137898942645300 | 212.85721902776600 | 0 | 0.019118656397892300 | 0.025052603743730700 | p_pajama_time_time_varying | PerProtocol_actual_use | PP | 12 | 54385 | 149 |
| **n_patients** | -1.92296664340648E-05 | 1.06805303353929E-06 | 324.158837318267 | 0 | -2.13230119133806E-05 | -1.7136320954749E-05 | p_pajama_time_time_varying | PerProtocol_actual_use | PP | 12 | 54385 | 149 |
| **average_patient_risk_score** | 0.05057197820480090 | 0.0030621069559334000 | 272.7590518524020 | 0 | 0.04457035885436190 | 0.056573597555240000 | p_pajama_time_time_varying | PerProtocol_actual_use | PP | 12 | 54385 | 149 |
| **ns(time_days, knots = 30)1** | 0.20742726530750700 | 0.0025138942453301200 | 6808.284191860820 | 0 | 0.20250012312571700 | 0.2123544074892960 | p_pajama_time_time_varying | PerProtocol_actual_use | PP | 12 | 54385 | 149 |
| **ns(time_days, knots = 30)2** | 0.14481483689175700 | 0.0015252432496430500 | 9014.629785060290 | 0 | 0.14182541505479400 | 0.14780425872872000 | p_pajama_time_time_varying | PerProtocol_actual_use | PP | 12 | 54385 | 149 |
| **(Intercept)** | -0.016082661247588400 | 0.002503636077660930 | 41.26419969195380 | 1.32982291844996E-10 | -0.020989697790199000 | -0.011175624704977900 | p_manual_composition_time_varying | PerProtocol_actual_use | PP | 12 | 54385 | 149 |
| **exposure_groupabridge** | 0.0034911941469661600 | 0.0017191464734228000 | 4.124034029656260 | 0.042278150793574800 | 1.21728974908416E-04 | 0.0068606593190239000 | p_manual_composition_time_varying | PerProtocol_actual_use | PP | 12 | 54385 | 149 |
| **exposure_groupscribe** | 0.04571761437170860 | 0.0013160365959816500 | 1206.7885687934900 | 0 | 0.04313823004124790 | 0.0482969987021693 | p_manual_composition_time_varying | PerProtocol_actual_use | PP | 12 | 54385 | 149 |
| **n_patients** | -1.42980576595863E-05 | 1.89650220626428E-06 | 56.839125005425400 | 4.72955008490317E-14 | -1.80151336804651E-05 | -1.05809816387076E-05 | p_manual_composition_time_varying | PerProtocol_actual_use | PP | 12 | 54385 | 149 |
| **average_patient_risk_score** | -0.05367104493811170 | 0.0022587735885979900 | 564.5919733625330 | 0 | -0.05809815982099410 | -0.049243930055229400 | p_manual_composition_time_varying | PerProtocol_actual_use | PP | 12 | 54385 | 149 |
| **ns(time_days, knots = 30)1** | 0.3365123138998040 | 0.002988970162000900 | 12675.31504337310 | 0 | 0.3306540400314180 | 0.34237058776819100 | p_manual_composition_time_varying | PerProtocol_actual_use | PP | 12 | 54385 | 149 |
| **ns(time_days, knots = 30)2** | 0.2204451965761200 | 0.0016540463097983700 | 17762.57607017540 | 0 | 0.2172033253801540 | 0.2236870677720860 | p_manual_composition_time_varying | PerProtocol_actual_use | PP | 12 | 54385 | 149 |
| **(Intercept)** | -0.20920224539130600 | 0.006343824171141500 | 1087.5028616588300 | 0 | -0.22163591229099800 | -0.19676857849161400 | p_visit_closed2days_time_varying | PerProtocol_actual_use | PP | 12 | 54385 | 149 |
| **exposure_groupabridge** | -0.027735175992053100 | 0.004003458811370080 | 47.99446142645650 | 4.27424762250439E-12 | -0.03558181107592800 | -0.019888540908178200 | p_visit_closed2days_time_varying | PerProtocol_actual_use | PP | 12 | 54385 | 149 |
| **exposure_groupscribe** | -0.12022355037874000 | 0.0027701083164699000 | 1883.586617007940 | 0 | -0.12565286291229600 | -0.11479423784518400 | p_visit_closed2days_time_varying | PerProtocol_actual_use | PP | 12 | 54385 | 149 |
| **n_patients** | 5.78506897182182E-06 | 3.56465883436822E-06 | 2.6337899773708000 | 0.10461173427388200 | -1.20153396071241E-06 | 1.27716719043561E-05 | p_visit_closed2days_time_varying | PerProtocol_actual_use | PP | 12 | 54385 | 149 |
| **average_patient_risk_score** | 0.008602995751460120 | 0.0053608931620311600 | 2.575283898219620 | 0.10854462621778700 | -0.001904161771088000 | 0.019110153274008200 | p_visit_closed2days_time_varying | PerProtocol_actual_use | PP | 12 | 54385 | 149 |
| **ns(time_days, knots = 30)1** | 1.4260264254501500 | 0.0073577538121683500 | 37563.37812668980 | 0 | 1.4116054929711900 | 1.4404473579291100 | p_visit_closed2days_time_varying | PerProtocol_actual_use | PP | 12 | 54385 | 149 |
| **ns(time_days, knots = 30)2** | 1.0028747271369400 | 0.0030204893783401100 | 110239.88420450600 | 0 | 0.9969546767397080 | 1.0087947775341700 | p_visit_closed2days_time_varying | PerProtocol_actual_use | PP | 12 | 54385 | 149 |
| **(Intercept)** | -0.3481123553460470 | 0.009368373670913380 | 1380.7354192237700 | 0 | -0.3664740303347510 | -0.3297506803573440 | opportunity_score_time_varying | PerProtocol_actual_use | PP | 12 | 54385 | 149 |
| **exposure_groupabridge** | -0.007250657653371170 | 0.005398708028488330 | 1.8037449100002400 | 0.17926041207501700 | -0.01783193095225550 | 0.003330615645513190 | opportunity_score_time_varying | PerProtocol_actual_use | PP | 12 | 54385 | 149 |
| **exposure_groupscribe** | -0.03541606804931640 | 0.0043365307626043700 | 66.69846263608620 | 3.33066907387547E-16 | -0.04391551216187100 | -0.02691662393676180 | opportunity_score_time_varying | PerProtocol_actual_use | PP | 12 | 54385 | 149 |
| **n_patients** | 3.5594063480152E-05 | 5.20397052934998E-06 | 46.782721706019000 | 7.93098919871227E-12 | 2.53944686660182E-05 | 4.57936582942858E-05 | opportunity_score_time_varying | PerProtocol_actual_use | PP | 12 | 54385 | 149 |
| **average_patient_risk_score** | -0.0017037447699267400 | 0.008244048955705040 | 0.042709840264202600 | 0.836272592892941 | -0.017861783809893600 | 0.014454294270040200 | opportunity_score_time_varying | PerProtocol_actual_use | PP | 12 | 54385 | 149 |
| **ns(time_days, knots = 30)1** | 1.9206795049941200 | 0.010353926659811200 | 34411.1855607241 | 0 | 1.9003861816423200 | 1.9409728283459200 | opportunity_score_time_varying | PerProtocol_actual_use | PP | 12 | 54385 | 149 |
| **ns(time_days, knots = 30)2** | 1.3307979618194800 | 0.004752664799783940 | 78406.10574887450 | 0 | 1.3214829099813100 | 1.340113013657650 | opportunity_score_time_varying | PerProtocol_actual_use | PP | 12 | 54385 | 149 |

**Supplementary Table 5 – Sensitivity**

| **term** | **estimate** | **std.error** | **statistic** | **p.value** | **conf.low** | **conf.high** | **outcome** | **analysis** | **protocol** | **window_months** | **n_rows** | **n_providers** |
| --- | --- | --- | --- | --- | --- | --- | --- | --- | --- | --- | --- | --- |
| **exposure_groupabridge** | 0.00885156647049555 | 0.005739234793348630 | 2.37865954107889 | 0.12300305152513300 | -0.0023971270232869400 | 0.02010025996427800 | opportunity_score_time_varying | Sensitivity_3m | ITT | 3 | 39653 | 149 |
| **exposure_groupabridge** | -0.00821095640909313 | 0.005787962456183580 | 2.0125008953959100 | 0.1560079616041570 | -0.019555154367082900 | 0.003133241548896670 | opportunity_score_time_varying | Sensitivity_6m | ITT | 6 | 46569 | 149 |
| **exposure_groupabridge** | -0.0050780059980168300 | 0.00554439344844017 | 0.8388384627693440 | 0.359729182458005 | -0.015944817473079400 | 0.005788805477045740 | opportunity_score_time_varying | Sensitivity_9m | ITT | 9 | 52410 | 149 |
| **exposure_groupabridge** | -0.007250657653371170 | 0.005398708028488330 | 1.8037449100002400 | 0.17926041207501700 | -0.01783193095225550 | 0.003330615645513190 | opportunity_score_time_varying | Sensitivity_12m | ITT | 12 | 54385 | 149 |
| **exposure_groupscribe** | -0.026396230205425600 | 0.004836890972773040 | 29.781818883938900 | 4.83505100401516E-08 | -0.03587636230920760 | -0.01691609810164350 | opportunity_score_time_varying | Sensitivity_3m | ITT | 3 | 39653 | 149 |
| **exposure_groupscribe** | 0.00519498707180885 | 0.004553790404507720 | 1.30143615293901 | 0.2539510603203980 | -0.003730278114170360 | 0.014120252257788100 | opportunity_score_time_varying | Sensitivity_6m | ITT | 6 | 46569 | 149 |
| **exposure_groupscribe** | -0.042128997119393300 | 0.004361388229568110 | 93.30668227193240 | 0 | -0.05067716097194370 | -0.03358083326684290 | opportunity_score_time_varying | Sensitivity_9m | ITT | 9 | 52410 | 149 |
| **exposure_groupscribe** | -0.03541606804931640 | 0.0043365307626043700 | 66.69846263608620 | 3.33066907387547E-16 | -0.04391551216187100 | -0.02691662393676180 | opportunity_score_time_varying | Sensitivity_12m | ITT | 12 | 54385 | 149 |
| **exposure_groupabridge** | 0.015444012935313200 | 0.0019030316720301500 | 65.86099398026470 | 4.44089209850063E-16 | 0.011714139396695100 | 0.019173886473931300 | p_manual_composition_time_varying | Sensitivity_3m | ITT | 3 | 39653 | 149 |
| **exposure_groupabridge** | 0.01189835700843340 | 0.0017921245983512000 | 44.079594393518600 | 3.15291126540274E-11 | 0.008385857339856700 | 0.015410856677010000 | p_manual_composition_time_varying | Sensitivity_6m | ITT | 6 | 46569 | 149 |
| **exposure_groupabridge** | 0.008389745328117090 | 0.0017497845006511700 | 22.989441742717700 | 1.62893570254674E-06 | 0.0049602307261343900 | 0.011819259930099800 | p_manual_composition_time_varying | Sensitivity_9m | ITT | 9 | 52410 | 149 |
| **exposure_groupabridge** | 0.0034911941469661600 | 0.0017191464734228000 | 4.124034029656260 | 0.042278150793574800 | 1.21728974908416E-04 | 0.0068606593190239000 | p_manual_composition_time_varying | Sensitivity_12m | ITT | 12 | 54385 | 149 |
| **exposure_groupscribe** | 0.017942017528594500 | 0.0014922988010280300 | 144.55428607171300 | 0 | 0.015017165624407200 | 0.020866869432781700 | p_manual_composition_time_varying | Sensitivity_3m | ITT | 3 | 39653 | 149 |
| **exposure_groupscribe** | 0.043715496918868900 | 0.001437564396287510 | 924.7327153780540 | 0 | 0.040897922476688300 | 0.046533071361049400 | p_manual_composition_time_varying | Sensitivity_6m | ITT | 6 | 46569 | 149 |
| **exposure_groupscribe** | 0.04491075823041470 | 0.0013506462616075600 | 1105.6496504616300 | 0 | 0.04226354020181020 | 0.04755797625901910 | p_manual_composition_time_varying | Sensitivity_9m | ITT | 9 | 52410 | 149 |
| **exposure_groupscribe** | 0.04571761437170860 | 0.0013160365959816500 | 1206.7885687934900 | 0 | 0.04313823004124790 | 0.0482969987021693 | p_manual_composition_time_varying | Sensitivity_12m | ITT | 12 | 54385 | 149 |
| **exposure_groupabridge** | -0.0021735468148145000 | 0.001189723311131880 | 3.3376904420090700 | 0.0677095863560997 | -0.004505361656200730 | 1.58268026571729E-04 | p_pajama_time_time_varying | Sensitivity_3m | ITT | 3 | 39653 | 149 |
| **exposure_groupabridge** | -0.004090356484559380 | 0.0013820966047963000 | 8.75881833382049 | 0.0030810861482243300 | -0.006799216053115220 | -0.0013814969160035500 | p_pajama_time_time_varying | Sensitivity_6m | ITT | 6 | 46569 | 149 |
| **exposure_groupabridge** | -0.008214468052808520 | 0.0017030716366265300 | 23.264464344938000 | 1.4118462006163E-06 | -0.011552427123688200 | -0.004876508981928840 | p_pajama_time_time_varying | Sensitivity_9m | ITT | 9 | 52410 | 149 |
| **exposure_groupabridge** | -0.007434806153230080 | 0.001797766889148230 | 17.103009798609900 | 3.54061502575442E-05 | -0.01095836450855920 | -0.003911247797900940 | p_pajama_time_time_varying | Sensitivity_12m | ITT | 12 | 54385 | 149 |
| **exposure_groupscribe** | 0.01645330622162200 | 0.001060265022384240 | 240.81164942715200 | 0 | 0.014375224963681400 | 0.018531387479562700 | p_pajama_time_time_varying | Sensitivity_3m | ITT | 3 | 39653 | 149 |
| **exposure_groupscribe** | 0.02612647542329360 | 0.0012588516782349300 | 430.7373426685470 | 0 | 0.023659171472075300 | 0.028593779374511800 | p_pajama_time_time_varying | Sensitivity_6m | ITT | 6 | 46569 | 149 |
| **exposure_groupscribe** | 0.020723473437498800 | 0.0015015713526049700 | 190.4728803726870 | 0 | 0.017780447666175900 | 0.023666499208821600 | p_pajama_time_time_varying | Sensitivity_9m | ITT | 9 | 52410 | 149 |
| **exposure_groupscribe** | 0.022085630070811500 | 0.0015137898942645300 | 212.85721902776600 | 0 | 0.019118656397892300 | 0.025052603743730700 | p_pajama_time_time_varying | Sensitivity_12m | ITT | 12 | 54385 | 149 |
| **exposure_groupabridge** | -0.012273046651618000 | 0.004470295318915440 | 7.53758704996483 | 0.006042491117933540 | -0.021034664476950300 | -0.003511428826285740 | p_visit_closed2days_time_varying | Sensitivity_3m | ITT | 3 | 39653 | 149 |
| **exposure_groupabridge** | -0.02683397331151900 | 0.004492549045867050 | 35.67667012970240 | 2.32936936406247E-09 | -0.03563920764019820 | -0.018028738982839800 | p_visit_closed2days_time_varying | Sensitivity_6m | ITT | 6 | 46569 | 149 |
| **exposure_groupabridge** | -0.020111335143979700 | 0.004243564749808030 | 22.460537252837900 | 2.14505498952189E-06 | -0.028428569219667100 | -0.011794101068292200 | p_visit_closed2days_time_varying | Sensitivity_9m | ITT | 9 | 52410 | 149 |
| **exposure_groupabridge** | -0.027735175992053100 | 0.004003458811370080 | 47.99446142645650 | 4.27424762250439E-12 | -0.03558181107592800 | -0.019888540908178200 | p_visit_closed2days_time_varying | Sensitivity_12m | ITT | 12 | 54385 | 149 |
| **exposure_groupscribe** | -0.07153179550246550 | 0.0033616762757594200 | 452.7792066210870 | 0 | -0.07812055993063660 | -0.06494303107429430 | p_visit_closed2days_time_varying | Sensitivity_3m | ITT | 3 | 39653 | 149 |
| **exposure_groupscribe** | -0.07598298118017860 | 0.0031919920065872600 | 566.6423994591310 | 0 | -0.08223917055202940 | -0.06972679180832790 | p_visit_closed2days_time_varying | Sensitivity_6m | ITT | 6 | 46569 | 149 |
| **exposure_groupscribe** | -0.11918339651811000 | 0.002895691349277960 | 1694.0529410793400 | 0 | -0.12485884727303900 | -0.11350794576318100 | p_visit_closed2days_time_varying | Sensitivity_9m | ITT | 9 | 52410 | 149 |
| **exposure_groupscribe** | -0.12022355037874000 | 0.0027701083164699000 | 1883.586617007940 | 0 | -0.12565286291229600 | -0.11479423784518400 | p_visit_closed2days_time_varying | Sensitivity_12m | ITT | 12 | 54385 | 149 |

**Supplementary Text**

**Statistical Code**

```{r setup, include=FALSE}

\# Load required packages

required_pkgs <- c("tidyverse", "tableone", "janitor", "openxlsx", "labelled", "scales", "forcats", "ggpubr", "geepack", "splines", "broom", "flextable", "officer")

for (pkg in required_pkgs) {

if (!requireNamespace(pkg, quietly = TRUE)) install.packages(pkg)

library(pkg, character.only = TRUE)

}

```

```{r load-clean}

\# Load and clean wide-format data

df_wide <- read_csv("provider_level_outcomes_.csv") %>%

clean_names() %>%

mutate(

exposure_group = factor(exposure_group, levels = c("none", "Tool A", "Tool B", "Tool C")),

exposure_group = recode(exposure_group,

"Tool A" = "Tool A - Tablet-Based, Human-Assisted Ambient Tool",

"Tool B" = "Tool B - Integrated Ambient Tool",

"Tool C" = "Tool C - Standalone/Multilingual Ambient Tool"

),

sex = factor(sex),

provider_type_cd = factor(provider_type_cd),

ethnicity = fct_explicit_na(ethnicity, na_level = "Missing"),

sequence = fct_explicit_na(sequence, na_level = "Missing"),

n_switches = as.numeric(n_switches),

time_to_first_exposure_days = as.numeric(time_to_first_exposure_days),

across(contains("date"), ~ as.character(.))

)

\# Labels for clarity

var_labels <- c(

sex = "Sex", ethnicity = "Ethnicity", age = "Provider Age", provider_type_cd = "Provider Type",

n_patients = "Number of Patients Seen", average_patient_risk_score = "Avg Patient Risk Score",

sequence = "Exposure Sequence", n_switches = "Number of Exposure Switches",

time_to_first_exposure_days = "Days to First Exposure", avg_pajama_time_outcome = "Avg Pajama Time",

avg_visit_closed_2days_outcome = "Visit Closed ≤2 Days", avg_manual_composition_outcome = "Manual Note Composition",

avg_opportunity_score_outcome = "Opportunity Score"

)

\# Define variables for Table 1

factorVars <- c("sex", "ethnicity", "provider_type_cd", "sequence")

numericVars <- c("age", "n_patients", "average_patient_risk_score", "n_switches", "time_to_first_exposure_days",

"avg_pajama_time_outcome", "avg_visit_closed_2days_outcome", "avg_manual_composition_outcome",

"avg_opportunity_score_outcome")

```

```{r table1-summary, message=FALSE, warning=FALSE}

\# ---- Table 1: Baseline Characteristics by Exposure Group ----

\# Create Table 1 using updated labels

var_labels <- c(

sex = "Sex",

ethnicity = "Ethnicity",

age = "Provider Age",

provider_type_cd = "Provider Type",

n_patients = "Patients Seen (Pre) per Provider-Day",

average_patient_risk_score = "Avg Patient Risk Score",

sequence = "Exposure Sequence",

n_switches = "Exposure Switches",

time_to_first_exposure_days = "Days to First Exposure",

avg_pajama_time_outcome = "Avg Pajama Time",

avg_visit_closed_2days_outcome = "Visit Closed ≤2 Days",

avg_manual_composition_outcome = "Manual Composition",

avg_opportunity_score_outcome = "Opportunity Score")

factorVars <- c("sex", "ethnicity", "provider_type_cd", "sequence")

numericVars <- c("age", "n_patients", "average_patient_risk_score", "n_switches",

"time_to_first_exposure_days", "avg_pajama_time_outcome",

"avg_visit_closed_2days_outcome", "avg_manual_composition_outcome",

"avg_opportunity_score_outcome", "avg_adjusted_wrvus_outcome", "avg_total_wrvus_outcome")

\# Create and print Table 1

summary_table1 <- CreateTableOne(vars = c(factorVars, numericVars),

strata = "exposure_group",

data = df_wide,

factorVars = factorVars,

includeNA = TRUE)

print(summary_table1, showAllLevels = TRUE, quote = TRUE, noSpaces = TRUE)

table1_df <- print(summary_table1, quote = FALSE, noSpaces = TRUE, printToggle = FALSE) %>%

as.data.frame() %>% rownames_to_column("Variable")

table1_df$Variable <- recode(table1_df$Variable, !!!var_labels)

write_csv(table1_df, "table1_ambient_baseline_characteristics.csv")

```

```{r prepost-summary, message=FALSE, warning=FALSE}

\# ---- Crude Pre/Post Comparison ----

\# Load long-format data

long_df <- read_csv("provider_long_df_.csv") %>%

clean_names() %>%

mutate(

rel_day = as.numeric(current_date - first_exposure_date),

pre_flag = rel_day < 0,

post_flag = rel_day >= 0

)

\# Define daily outcomes

outcome_vars <- c(

"p_pajama_time_time_varying",

"p_manual_composition_time_varying",

"p_visit_closed2days_time_varying",

"opportunity_score_time_varying"

)

\# Create pre vs post comparisons by provider and exposure group

prepost_summary <- long_df %>%

pivot_longer(cols = all_of(outcome_vars), names_to = "outcome", values_to = "value") %>%

filter(pre_flag | post_flag) %>%

mutate(period = if_else(pre_flag, "pre", "post")) %>%

group_by(EHR_provider_id, exposure_group, outcome, period) %>%

summarise(mean_value = mean(value, na.rm = TRUE), .groups = "drop") %>%

pivot_wider(names_from = period, values_from = mean_value) %>%

mutate(diff = post - pre)

\# Summarize across providers by group

prepost_summary_group <- prepost_summary %>%

group_by(exposure_group, outcome) %>%

summarise(

n = n(),

mean_pre = mean(pre, na.rm = TRUE),

mean_post = mean(post, na.rm = TRUE),

mean_diff = mean(diff, na.rm = TRUE),

sd_diff = sd(diff, na.rm = TRUE),

.groups = "drop"

)

write_csv(prepost_summary_group, "table2_prepost_crude_comparison.csv")

```

```{r gee-models, message=FALSE, warning=FALSE}

\# ---- Comparative Effectiveness Models (GEE) ----

library(geepack)

library(splines)

library(broom)

\# Prepare GEE-ready dataset

gee_df <- long_df %>%

filter(exposure_group != "none") %>%

mutate(exposure_group = fct_relevel(factor(exposure_group), "Tool B"))

\# Define outcomes and model formulas

outcomes <- c(

"p_pajama_time_time_varying",

"p_manual_composition_time_varying",

"p_visit_closed2days_time_varying",

"opportunity_score_time_varying"

)

formulas <- list(

crude	= "~ exposure_group",

volume	= "~ exposure_group + n_patients",

risk	= "~ exposure_group + average_patient_risk_score",

full	= "~ exposure_group + n_patients + average_patient_risk_score",

spline30 = "~ exposure_group + n_patients + average_patient_risk_score + ns(time_days, knots = 30)"

)

all_results <- list()

\# Fit GEE models

for (y in outcomes) {

for (m in names(formulas)) {

fml <- as.formula(paste0(y, formulas[[m]]))

mod <- tryCatch(

geeglm(fml, id = EHR_provider_id, data = gee_df,

family = gaussian, corstr = "exchangeable"),

error = function(e) NULL

)

if (!is.null(mod)) {

all_results[[paste(y, m, sep = "_")]] <- tidy(mod, conf.int = TRUE) %>%

mutate(model = m, outcome = y)

}

}

}

\# Save combined results

df_all_models <- bind_rows(all_results)

write_csv(df_all_models, "gee_results_all_models.csv")
